# Secreted Protein VdCUE Modulates Virulence of *Verticillium dahliae* Without Interfering with BAX-Induced Cell Death

**DOI:** 10.3390/jof11090660

**Published:** 2025-09-08

**Authors:** Haonan Yu, Haiyuan Li, Xiaochen Zhang, Mengmeng Wei, Xiaoping Hu, Jun Qin

**Affiliations:** 1 State Key Laboratory for Crop Stress Resistance and High-Efficiency Production, College of Plant Protection, Northwest A&F University, Yangling 712100, China; yuhaonan623@163.com (H.Y.); zhangxiaochen1209@163.com (X.Z.); weimengmeng212@163.com (M.W.); 2 Institute of Plant Protection, Jiangxi Academy of Agricultural Sciences, Nanchang 330200, China; lihai-yuan@jxaas.cn

**Keywords:** *Verticillium dahliae*, secreted protein, microsclerotia, virulence, programmed cell death

## Abstract

Verticillium wilt, caused by *Verticillium dahliae*, severely threatens various crops and trees worldwide. This study aimed to characterize the function of a CUE (coupling of ubiquitin conjugation to endoplasmic reticulum (ER) degradation)-domain-containing protein, VdCUE, in *V. dahliae*, which exhibits sequence divergence between the defoliating strain XJ592 and the non-defoliating strain XJ511. We generated ∆*VdCUE*-knockout mutants and evaluated their phenotypes in growth and virulence. Functional analyses included verifying the signal peptide activity of VdCUE, testing its ability to induce cell death or inhibit *BAX*-induced cell death in *Nicotiana benthamiana* leaves, and identifying host targets via yeast two-hybrid screening. The ∆*VdCUE* mutants showed reduced formation of melanized microsclerotia but no other obvious growth defects. Cotton plants infected with the ∆*VdCUE* mutants exhibited a significantly lower disease index and defoliation rate. VdCUE was confirmed to be secreted via a functional signal peptide, but it neither triggered cell death nor inhibited *BAX*-induced cell death. Three putative host targets were identified and supported by AI-based three-dimensional structural modeling, including tRNA-specific 2-thiouridylase, peptidyl-prolyl cis-trans isomerase, and 40S ribosomal protein, which may mediate VdCUE-dependent virulence regulation. These findings reveal VdCUE as a key virulence factor in *V. dahliae*, contributing to our understanding of its pathogenic mechanism.

## 1. Introduction

Verticillium wilt caused by *Verticillium dahliae* is a destructive soil-borne disease affecting a broad range of crops and trees, including cotton, potato, lettuce, eggplant, olive tree, and smoke tree, leading to reduced crop yields and substantial economic losses worldwide [1,2,3]. The dormant structure of *V. dahliae*, the microsclerotia, can survive in soil for more than 10 years, thereby making Verticillium wilt difficult to control [4]. Currently, breeding new disease-resistant varieties remains the most effective approach for controlling this disease. Therefore, uncovering more virulence factors in pathogens and identifying more resistance or susceptibility genes in host plants are critical strategies for offering candidate targets for disease-resistance breeding.

Employing effectors is generally a strategy for phytopathogens to combat host immunity. Similarly, as a soil-borne pathogen, *V. dahliae* also secretes a large number of effector proteins to conquer various disease resistance mechanisms of plants in order to successfully colonize in plant xylem vessels [5]. Due to the subcellular localization in host plants, effectors can be divided into cytoplasmic effectors and apoplastic effectors. VdCE11 has been identified to be a cytoplasmic effector, where it interacts with cotton aspartic protease GhAP1 and promotes its accumulation, leading to an increased susceptibility of cotton to *V. dahliae* [6]. The secreted feruloyl esterase VdFAE is capable of triggering cell death in *Nicotiana benthamiana* leaves, while upregulating defense-related genes and enhancing resistance to *Pseudomonas syringae* pv. *tomato* DC3000 [7]. In addition, during the infection of cotton by *V. dahliae*, VdFAE exerts a virulence function by directly binding to and promoting the degradation of a disease resistance protein, GhDFR [7]. Among cytoplasmic effectors, a subset directly targets the host nucleus to exert its functions. For instance, VdSCP41 targets nucleus-localized transcription factors CBP60g and SARD1 to disrupt their transcription activity, resulting in reduced expression of downstream defense-related genes and impaired cotton immunity [8]. *Verticillium*-specific protein VdSCP7 targets the host nucleus to induce host immunity by activating salicylic acid (SA) and jasmonic acid (JA) signaling pathways [9]. Apoplast effectors also play irreplaceable roles in regulating pathogen virulence and host immunity. One is the endopolygalacturonase VdEPG1, which translocates to the host cell membrane to target the positive regulator of disease resistance, GhOPR9, and suppress host immunity [10]. Some apoplast effectors, such as secreted small cysteine-rich proteins (SCPs) VdSCP27, VdSCP113, VdSCP126, and ethylene-inducing xylanase (EIX)-like protein VdEIX3, however, act as the inducers of immune responses in *N. benthamiana* leaves, including cell death, oxidative burst, and the upregulation of defense-related genes [11,12]. Nowadays, an increasing number of novel effector proteins are being identified, but only when their functional mechanisms are elucidated can they be utilized for disease control.

Ubiquitination is a highly regulated post-translational modification in eukaryotic cells; it is catalyzed by three enzymes, ubiquitin-activating enzyme (E1), ubiquitin-conjugating enzyme (E2), and ubiquitin ligase (E3), that covalently attach ubiquitin to target proteins. Ubiquitinated proteins are then either recognized and degraded by the 26S proteasome or functionally regulated in processes such as signal transduction or endocytosis [13]. ERAD (*Endoplasmic reticulum* (ER)-associated degradation) is a protein quality control mechanism, where misfolded or unassembled proteins are ubiquitinated by ER membrane-localized E2 and E3 enzymes, retrotranslocated to the cytoplasm, and degraded by the 26S proteasome [14]. The CUE (coupling of ubiquitin conjugation to ER degradation) domain, which typically consists of approximately 40 amino acids and forms a three-helical bundle structure, binds to ubiquitin and facilitates the elongation of ubiquitin chains during ERAD [15,16].

Cue1p, one of the best-characterized CUE-domain-containing proteins in budding yeast, not only functions in ubiquitin binding but also mediates the recruitment of Ubc7 to the ER membrane [16]. Cue2 endonuclease harbors three CUE domains and plays a role in mRNA cleavage by either recognizing the polyubiquitination of ribosomal protein eS7 or locating within the colliding ribosome [17]. Two other CUE-domain-containing proteins in yeast, Vps9p and Vps901p play a role in vesicle-mediated transport and localization of vacuolar proteins [18,19]. The CUE domain is also of significant importance in plants. RIN2 and RIN3 are two CUE-domain-containing proteins in *Arabidopsis thaliana*, which positively regulate hypersensitive response mediated by RPM1 and RPS2 [20]. *A. thaliana* CUE protein variant IPD1 regulates hypocotyl elongation dependent on blue and far-red light [21]. In rice, *SPL35* (*Spotted leaf 35*) encodes a CUE-domain-containing protein, the disruption of which enhances plant immunity with higher H_2_O_2_ accumulation and defense gene expression [22].

Among plant pathogens, functional characterization of the CUE domain remains limited. *MoCUE1* encodes a CUE-domain-containing protein in *Magnaporthe oryzae*, the causal agent of destructive rice blast, and has been identified as the ortholog of yeast Cue1p [23]. Deletion of *MoCUE1* reduces mycelial growth and conidiation, impairs the ER stress response, and decreases both the accumulation of certain effectors in the biotrophic interfacial complex (BIC) and fungal virulence [23]. However, the functions of CUE domains and their underlying molecular mechanisms in plant pathogenic fungi remain largely unknown, highlighting the need for more in-depth investigations.

In our previous study, the genomes of the defoliating strain XJ592 and the non-defoliating strain XJ511 of *V. dahliae* were sequenced [24]. From the sequencing data, a secreted protein designated VdCUE (Table S2) was identified; it exhibits sequence differences between the two strains, suggesting its potential involvement in virulence and defoliation pathotype [24]. To uncover its roles in the *V. dahliae*–cotton interaction, *VdCUE* was knocked out in XJ592. The knockout mutants were evaluated for growth rate, phenotype, virulence, and cotton defoliation rate. The ability of *VdCUE* to regulate plant programmed cell death was also tested. Furthermore, three host targets of VdCUE in cotton were identified and analyzed using AI-based three-dimensional structural modeling. Our findings will expand the understanding of the mechanisms by which phytopathogens utilize secreted proteins to modulate virulence and advance the development of targeted disease control strategies for Verticillium wilt.

## 2. Materials and Methods

### 2.1. Fungal Strains, Plant Materials, and Culture Conditions

The defoliating *V. dahliae* strain XJ592 was isolated from diseased cotton plants in Xinjiang Province, China, and preserved at −80 °C in 25% glycerol. Fungal strains were cultured on Potato Dextrose Agar (PDA) in the dark at 25 °C. For virulence evaluation, the susceptible cotton cultivar ‘Jimian 11’ was employed. Cotton seeds were sown in pots filled with a vermiculite–substrate mixture (1:2 ratio) and cultivated in a greenhouse at 25 °C under a 16 h light/8 h dark photoperiod. *N. benthamiana* was grown under the same conditions as ‘Jimian 11’.

### 2.2. Deletion and Complementation of VdCUE in XJ592

The 1.5 kb upstream and downstream flanking sequences of *VdCUE* were amplified from the genomic DNA of XJ592 and ligated into the gene-knockout vector pGKO-HPT, generating the deletion construct pGKO-*VdCUE* [25]. This recombinant plasmid was transformed into *Agrobacterium tumefaciens* strain EHA105, which was then used to transform XJ592 via *A. tumefaciens*-mediated transformation (ATMT). Transformants were selected on PDA plates supplemented with appropriate antibiotics. After 5–7 days of incubation, PCR screening was performed to identify the correct deletion mutants. Two independent mutant strains, Δ*VdCUE-20* and Δ*VdCUE-21*, were selected for subsequent experiments. The primers used in PCR are listed in Table S1.

For genetic complementation, the full-length coding sequence (CDS) of *VdCUE* was amplified from XJ592 cDNA and cloned into the vector pSul-2k-GFP, generating the complementation construct pSul-2k-*VdCUE*-GFP. This plasmid was transformed into *A. tumefaciens* EHA105, and the resulting strain was used to generate the complemented strain Δ*VdCUE*-C via ATMT. Correct integration of the complementation construct was verified by PCR.

### 2.3. Growth Rate and Microsclerotia Formation Assays

Mycelial plugs of identical diameter were excised from the wild-type strain XJ592, gene deletion mutants Δ*VdCUE-20* and Δ*VdCUE-21*, and the complemented strain Δ*VdCUE*-C. The plugs were inoculated at the center of PDA plates, which were then cultured at 25 °C in the dark. Following 2 weeks post inoculation (wpi), colony morphology was photographed, and colony diameter was measured using the cross-diameter method. Meanwhile, the fungal colonies were observed to check the production of microsclerotia by visual inspection. The experiment was performed with three replicate plates for each strain, and the entire assay was repeated three times independently.

### 2.4. Virulence Assessment Assays

Mycelial plugs from the wild-type and mutant strains were inoculated into CM liquid medium (6 g/L yeast extract, 6 g/L casein acid hydrolysate, and 10 g/L sucrose) and incubated with shaking at 25 °C and 160 rpm for 3 days. Following filtration with filter cloth, the conidial suspension was centrifuged at 5000 rpm for 5 min to pellet the conidia. The supernatant was discarded, and the conidial pellet was resuspended in sterile water. Uniform two-leaf stage cotton seedlings were inoculated via root dipping, with 50 mL of the prepared conidial suspension (1 × 10^7^ conidia/mL) poured directly into the soil around each seedling from the base. To ensure effective absorption by the roots, watering was withheld for three days prior to infection. Sterile water-inoculated seedlings served as negative controls. Three independent replicate experiments were performed, and in each experiment, each strain was inoculated onto 24–30 cotton seedlings of uniform growth status. The nutrient pots were placed in a greenhouse maintained at 25 °C under a 16 h light/8 h dark photoperiod for 28 days. The disease grade was classified as follows: 0 (no symptoms), 1 (0–25% wilted leaves), 2 (26–50% wilted leaves), 3 (51–75% wilted leaves), and 4 (76–100% wilted leaves) [6].

### 2.5. Sequence Analysis

Signal peptide (SP) prediction was performed using SignalP 5.0 (https://services.healthtech.dtu.dk/services/SignalP-5.0/, accessed on 1 July 2021), with the full-length amino acid sequence of VdCUE as input, and the eukaryotic prediction mode was selected (consistent with the biological property of *V. dahliae*). Domain alignment was performed using the SMART database (http://smart.embl.de/, accessed on 1 July 2021) and NCBI CD Search (https://www.ncbi.nlm.nih.gov/Structure/cdd/wrpsb.cgi, accessed on 1 July 2021), with an E-value threshold set to 0.01 to screen for significantly matched domains.

### 2.6. Yeast Secretion Assays

The pSUC2 vector encodes invertase but lacks an SP sequence. Only yeast strains transformed with a functional SP can secrete invertase and grow on YPRAA medium containing raffinose as the sole carbon source [26]. The predicted SP-coding sequence of *VdCUE* was inserted into the pSUC2 vector. The recombinant vector carrying the *VdCUE* SP was then transformed into yeast strain YTK12. The yeast transformed with the pSUC2 vector containing the Avr1b (protein accession number: AAM20936.1) SP served as the positive control, while the yeast transformed with the empty pSUC2 vector served as the negative control.

Positive colonies were first selected on CMD-W medium (6.7 g/L Yeast Nitrogen Base without Amino Acids, 0.74 g/L -Trp DO supplement, 20 g/L sucrose, 1 g/L glucose, and 20 g/L agar) at 30 °C for 3 days and then cultured on YPRAA medium (10 g/L yeast extract, 20 g/L peptone, 20 g/L raffinose, and 20 g/L agar) to assay invertase secretion. Invertase activity was detected using the 2,3,5-triphenyltetrazolium chloride (TTC) color reaction, in which invertase hydrolyzes sucrose into glucose and fructose. Colorless TTC is reduced to form red formazan crystals [27], with the intensity of the red color being proportional to invertase activity, serving as an indicator of relative activity levels rather than providing precise numerical quantification.

### 2.7. Agroinfiltration Assays

The recombinant vector was constructed using the *N. benthamiana* transient expression vector pBin-eGFP and subsequently transformed into *A. tumefaciens* strain GV3101. The transformed GV3101 strains harboring the relevant vectors were inoculated into liquid LB medium supplemented with appropriate antibiotics and cultured overnight at 28 °C with shaking. The *A. tumefaciens* GV3101 cells were then harvested and resuspended in infiltration buffer (10 mM MgCl_2_, 0.5 mM MES, and 0.2 mM acetosyringone) to a final optical density of OD_600_ = 0.5. For co-expression experiments, two cultures carrying respective vectors were mixed at a 1:1 ratio (each adjusted to OD_600_ = 0.5), followed by incubation in the dark at 28 °C for 2–3 h. The pre-incubated *Agrobacterium* suspension was then infiltrated into the abaxial side of 4-week-old *N. benthamiana* leaves using a needleless syringe. GFP and pro-apoptotic Bcl-2-associated X protein (BAX, protein accession number: NP_031553.1) were used as negative and positive controls, respectively.

*N. benthamiana* leaves were monitored at 5–7 days post-infiltration. The typical cell death phenotype in *N. benthamiana* leaves is the formation of pale brown dots, which gradually expand into irregular dark brown necrotic plaques before gradually turning withered, white, and brittle and are accompanied by slight shrinkage. Three independent replicate experiments were conducted, with each replicate containing three *N. benthamiana* plants.

### 2.8. Yeast Two-Hybrid (Y2H) Screening for Interaction Targets

The coding sequence of *VdCUE* without the signal peptide was cloned into the pGBKT7 vector to generate the bait vector. The mRNA from *V. dahliae*-infected *G. hirsutum* was used to construct a cDNA library in the prey vector pGADT7. The Y2H Gold yeast strain harboring the bait plasmid was inoculated into SD-Trp selective medium and cultured at 30 °C until the optical density reached OD_600_ ≈ 0.8. After centrifugation and resuspension, the yeast cells were mixed with the AD library solution at a 1:5 ratio in a 2 L Erlenmeyer flask. Then, 2 × YPDA medium supplemented with kanamycin was added, and the mixture was co-incubated at 30 °C with gentle shaking (30 rpm) for 20–24 h to facilitate mating. Following incubation, the cells were harvested, resuspended in 0.9% NaCl solution containing kanamycin, and plated onto SD-Trp-Leu-His + X-α-Gal+AbA selection plates for primary screening of blue colonies. Positive colonies were subsequently transferred to SD-Trp-Leu-His-Ade + X-α-Gal+AbA plates for secondary verification. Finally, confirmed clones were selected for sequencing, and potential interacting targets were analyzed via NCBI BLAST alignment (https://blast.ncbi.nlm.nih.gov/Blast.cgi, accessed on 10 December 2024).

### 2.9. Y2H-AOS

Three-dimensional structural modeling of proteins was performed using AlphaFold2 software v2.3.1 [28], followed by protein–protein docking with HDOCK software v1.1 [29,30] to obtain multiple protein–protein complex structures. These structures were sorted and screened based on their respective confidence scores. The protein–protein complex structure with the highest confidence score was selected as the target and imported into Pymol software v3.1 to analyze the protein–protein binding sites. According to the HDOCK Server (http://hdock.phys.hust.edu.cn/, accessed on 20 July 2025), a confidence score above 0.7 indicates a high likelihood of binding between the two molecules, a score between 0.5 and 0.7 suggests a possible binding interaction, and a score below 0.5 implies a low probability of binding.

### 2.10. Statistical Analysis

Data are presented as mean ± standard deviation (SD). Prior to conducting the Student’s *t*-test, we performed normality and homoscedasticity tests on all data used for the *t*-test. *p*-values greater than 0.05 indicate that the data in each group conformed to a normal distribution and the variances among groups were homogeneous. Statistical significance was determined via Student’s *t*-test using GraphPad Prism 8.0 software, with *p* < 0.05, *p* < 0.01, and *p* < 0.001 considered statistically significant and denoted as *, **, and *** in the figures. ns indicates no significant difference (*p* > 0.05).

## 3. Results

### 3.1. VdCUE Regulates Microsclerotia Formation but Not Growth in V. dahliae

A gene with a CUE domain, designated *VdCUE*, was identified in the genome of *V. dahliae* strain XJ592. To investigate the function of *VdCUE*, deletion and complementation mutant strains of *VdCUE* were constructed via the ATMT system. Two deletion strains (Δ*VdCUE-20* and Δ*VdCUE-21*) and the complemented strain Δ*VdCUE*-C were PCR-confirmed and utilized for subsequent experiments.

**Figure 1:**
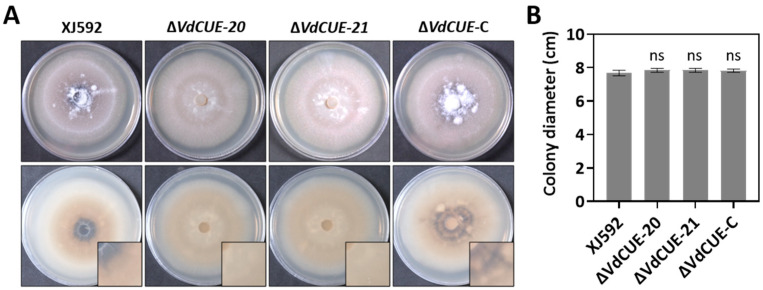
*VdCUE* promotes melanized microsclerotia formation but does not affect mycelial growth in *V. dahliae.* (**A**) Colony morphology of the wild-type strain XJ592, Δ*VdCUE-20*, Δ*VdCUE-21*, and Δ*VdCUE*-C strains cultured on PDA at 2 wpi. The images in the bottom right corner were 2.5× magnification of a quarter of each colony where melanized microsclerotia formed. (**B**) Colony diameters of the corresponding strains on PDA at 2 wpi. ns, no significant difference compared with the XJ592 strain (*p* > 0.05, Student’s *t*-test). The bar chart shows mean values and standard deviations derived from 3 independent replicates.

Mycelial plugs of identical diameter were excised from the wild-type strain XJ592, gene deletion mutants Δ*VdCUE-20* and Δ*VdCUE-21*, and the complemented strain Δ*VdCUE*-C. Each plug was individually inoculated onto the center of PDA plates and incubated in the dark at 25 °C for 2 weeks. Analysis of colony morphology showed that, unlike the XJ592 and Δ*VdCUE*-C strains, the Δ*VdCUE-20* and Δ*VdCUE-21* strains produced white mycelia with significantly reduced melanized microsclerotia (Figure 1A). No significant differences in colony diameter were observed among these strains (Figure 1B). These findings demonstrate that *VdCUE* is involved in microsclerotia development but is not required for the vegetative growth of *V. dahliae*.

### 3.2. VdCUE Positively Regulates Virulence and Cotton Defoliation in Strain XJ592

**Figure 2:**
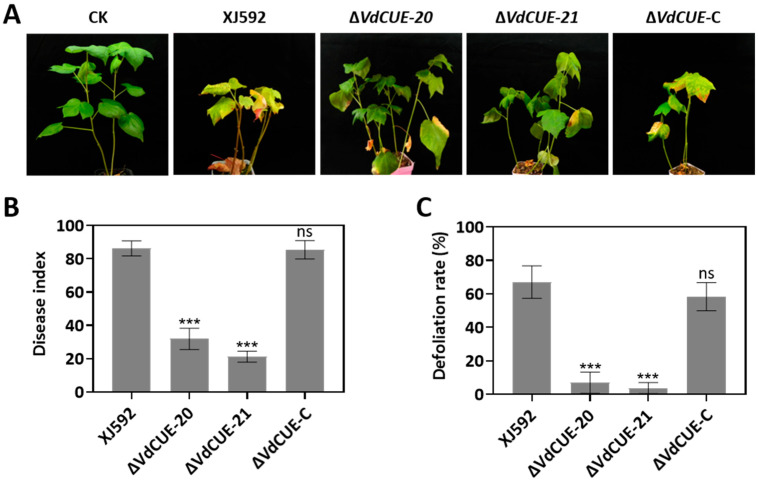
*VdCUE* positively regulates virulence and enhances the defoliating ability of *V. dahliae* XJ592. (**A**) Disease symptoms of cotton seedlings inoculated with the wild-type strain XJ592, Δ*VdCUE-20*, Δ*VdCUE-21*, and Δ*VdCUE*-C strains. Two-leaf-stage cotton seedlings were inoculated, and disease symptoms were observed at 4 wpi. Cotton seedlings inoculated with sterile water served as negative controls. (**B**) Disease index of cotton plants inoculated with the corresponding strains at 4 wpi. (**C**) Defoliation rate of cotton plants inoculated with the corresponding strains at 4 wpi. Error bars represent the standard deviations derived from three independent biological replicates, and all experiments were repeated three times with similar results. ***, *p* < 0.001 compared with the XJ592 strain (Student’s *t*-test). ns, no significant difference compared with the XJ592 strain (*p* > 0.05, Student’s *t*-test).

To investigate the role of *VdCUE* in the virulence of *V. dahliae* XJ592 and the leaf defoliation of inoculated cotton, conidia of XJ592, Δ*VdCUE-20*, Δ*VdCUE-21*, and Δ*VdCUE*-C were inoculated into two-leaf-stage susceptible cotton cultivar ‘Jimian 11’ via root-dip inoculation. Disease symptoms were assessed in cotton seedlings at 4 wpi. Cotton plants inoculated with XJ592 and Δ*VdCUE*-C showed typical Verticillium wilt symptoms, including leaf yellowing and wilting; in severely diseased plants, a significant proportion of leaves underwent defoliation. By contrast, cotton plants inoculated with Δ*VdCUE-20* and Δ*VdCUE-21* exhibited milder disease symptoms (Figure 2A and Figure S1A). Consistently, Δ*VdCUE-20* and Δ*VdCUE-21* caused significantly reduced disease index and defoliation rates on cotton plants compared with XJ592 and the complemented strain (Figure 2B,C and Figure S1B,C). These results indicate that *VdCUE* is critical for the virulence of XJ592.

### 3.3. VdCUE Contains a Functional SP and a CUE Domain

**Figure 3:**
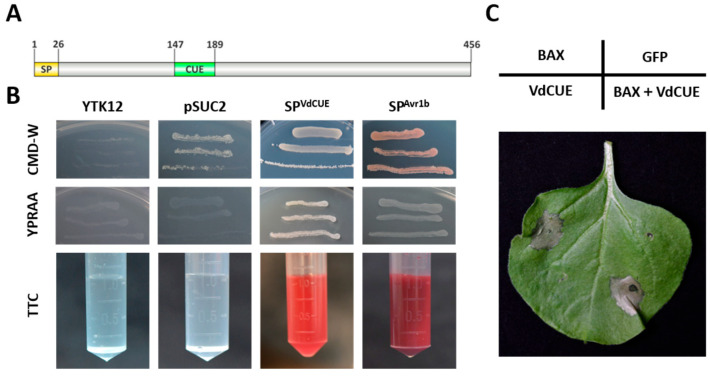
VdCUE contains an SP and a CUE domain and does not induce programmed cell death. (**A**) Schematic diagram of the *VdCUE* domain structure. (**B**) Yeast invertase secretion assay performed to characterize the SP-mediated secretion function of VdCUE. The yeast strain YTK12 was transformed with the empty vector pSUC2 (negative control), pSUC2-SP^Avr1b^ (positive control), or pSUC2-SP^VdCUE^. Transformants were assessed for growth on CMD-W and YPRAA media, and invertase activity was analyzed via the TTC chromogenic reaction. (**C**) Expression of *VdCUE* in *N. benthamiana* fails to trigger plant cell death and does not interfere with *BAX*-mediated cell death. Four-week-old plants were infiltrated with *A. tumefaciens* harboring pBin vectors for transient expression of GFP (negative control), *VdCUE*-GFP, *BAX* (positive control), and *VdCUE*-GFP with *BAX* (co-infiltrated).

VdCUE consists of 456 amino acids. Protein sequence analysis using SMART and SignalP-5.0 websites revealed that VdCUE contains both an SP and a CUE domain (Figure 3A). The CUE domain exhibits ubiquitin-binding activity. To date, few reports have described the function of CUE-domain-containing proteins in the pathogenic process of *V. dahliae*. In *Magnaporthe oryzae*, however, such proteins are involved in cellular ubiquitination processes and are closely associated with the pathogen’s virulence [23].

To verify the secretory function of the VdCUE SP (SP^VdCUE^), a secretion assay was conducted using yeast. The yeast strain YTK12 is deficient in invertase and thus unable to utilize oligosaccharides such as raffinose. The pSUC2 vector harbors invertase but lacks an SP sequence; therefore, only yeast strains transformed with a functional SP can secrete invertase normally and grow on YPRAA medium, where raffinose serves as the sole carbon source.

In this assay, the pSUC2 vector containing the Avr1b SP (SP^Avr1b^) sequence was used as the positive control, while YTK12 transformed with the empty pSUC2 vector served as the negative control. As expected, only the transformants carrying SP^VdCUE^ and the positive control strains exhibited normal growth on YPRAA medium (Figure 3B).

Furthermore, a TTC chromogenic assay confirmed the secretion of invertase by both SP^VdCUE^-harboring transformants and SP^Avr1b^-containing strains, as evidenced by the production of the red insoluble product 1,3,5-triphenylformazan (TPF). In contrast, the negative control strains neither grew on YPRAA medium nor induced a color change in the TTC assay (Figure 3B).

### 3.4. VdCUE Is Not Involved in the Regulation of Plant Programmed Cell Death

To investigate the role of *VdCUE* in plant immune responses, we generated a *VdCUE*-GFP fusion construct using the pBin-GFP vector. This construct was then transformed into *A. tumefaciens* GV3101, and the resulting strain was used to infiltrate *N. benthamiana* leaves for transient expression. GFP-expressing leaves served as the negative control, while the leaves expressing *BAX* were used as the positive control. No cell death was observed in the epidermal cells of *N. benthamiana* leaves expressing *VdCUE* alone, indicating that *VdCUE* itself lacks the activity to directly induce cell death. Furthermore, co-expression of *VdCUE* with *BAX* did not significantly suppress *BAX*-triggered hypersensitive response (HR) (Figure 3C and Figure S2), suggesting that *VdCUE* is not involved in regulating the *BAX*-induced plant programmed cell death.

### 3.5. Three Potential VdCUE-Interacting Proteins Were Identified by Y2H Screening

**Figure 4:**
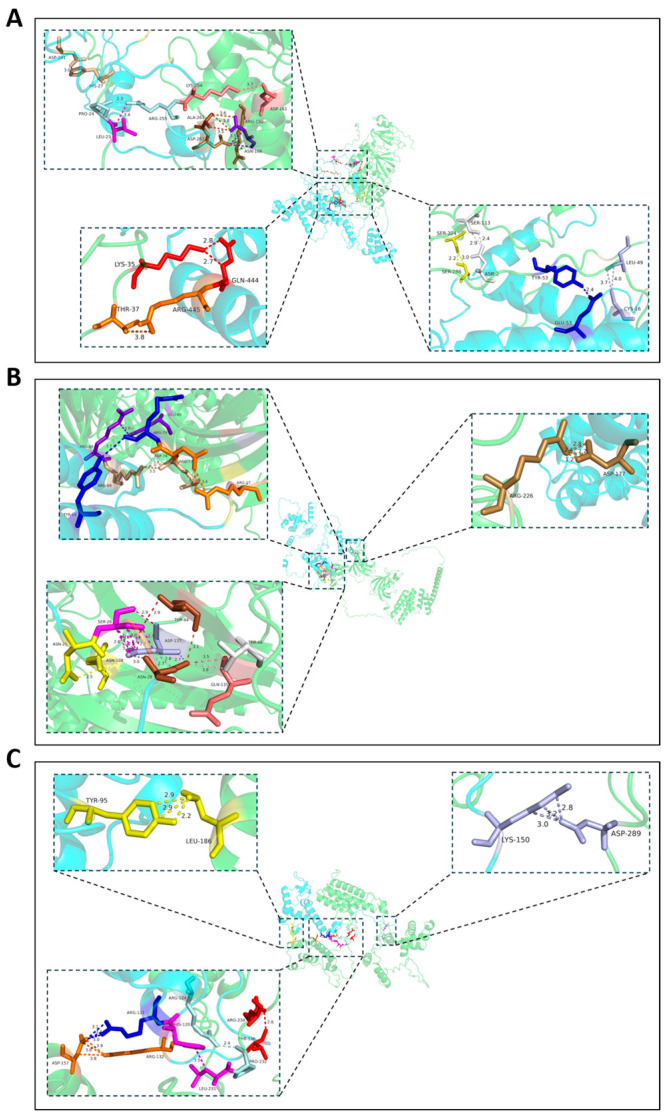
Three-dimensional structures of VdCUE and its target proteins, and their binding sites. (**A**) Three-dimensional structure and binding site of VdCUE and LOC107959305 interaction. (**B**) Three-dimensional structure and binding site of VdCUE and LOC107953988 interaction. (**C**) Three-dimensional structure and binding site of VdCUE and LOC107962302 interaction. The protein with light blue color represents VdCUE in each panel, while the protein with green color represents the target protein. Amino acids with the same color in each magnified image indicate predicted interacting pairs.

To dissect the molecular mechanisms underlying *VdCUE*-mediated virulence in *V. dahliae*, we performed a Y2H screen using VdCUE as bait to identify interacting proteins in cotton. This screen yielded three candidate targets: tRNA-specific 2-thiouridylase, peptidyl-prolyl cis-trans isomerase, and 40S ribosomal protein (Table 1). The three-dimensional structural modeling of protein–protein docking was then performed using AlphaFold2 and HDOCK software. Results supported the interaction between VdCUE and each of its host targets (confidence score above 0.7). The binding sites of protein pairs VdCUE-LOC107959305, VdCUE-LOC107953988, and VdCUE-LOC107962302 were analyzed, and the confidence scores were 0.9471, 0.8346, and 0.9301, respectively (Figure 4). In summary, VdCUE is likely to interact with these host targets to regulate the virulence of *V. dahliae*.

## 4. Discussion

*VdCUE*, encoding a CUE-domain-containing protein, has been demonstrated to contribute to the virulence of *V. dahliae* in this study. Since hyphal growth was unimpaired, a defect in microsclerotium development may be one of the factors underlying the attenuation of virulence in the *VdCUE*-knockout mutants. Given that VdCUE harbors a signal peptide that facilitates secretion, the interaction between VdCUE and host targets could represent another potential mechanism underlying virulence regulation.

Microsclerotia are specialized hyphal structures composed of densely packed, thick-walled hyphal cells, and they are generally black or dark brown. Melanized microsclerotia endow *V. dahliae* with the ability to tolerate various stress environments and survive for longer periods [31]. As the primary inoculum source, melanized microsclerotia are also associated with the virulence of *V. dahliae*. Numerous genes have been functionally characterized as regulators in the development of melanized microsclerotia. The C2H2-type transcription factor VdCf2 plays a negative role in melanin biosynthesis and a positive role in hyphal growth and virulence, without affecting the formation of microsclerotia [32]. In contrast to VdCf2, two other C2H2-type zinc finger proteins, VdZFP1 and VdZFP2, play a positive role in melanin biosynthesis and regulate the development and maturation of microsclerotia, as evidenced by the production of more and smaller microsclerotia than those of the wild type in their individual knockout mutants [33]. Notably, the virulence of the two mutants remains unaffected despite the delayed formation of melanized microsclerotia, suggesting that the timing of melanized microsclerotia formation is not strongly correlated with virulence [33]. Like *VdZFP1* and *VdZFP2* mutants, the *VdNuo1* (which encodes the 24 kDa subunit of mitochondrial complex I)-knockout mutants also produce more and smaller microsclerotia, but their virulence is significantly reduced [34]. The ∆*VdCUE* mutants produced fewer melanized microsclerotia, with no other obvious growth defects. The reduced virulence of ∆*VdCUE* mutants may be partly due to their defect in the formation of melanized microsclerotia. Moreover, in contrast to the genes described above, *VdCUE* encodes a secreted protein, which endows VdCUE with the potential to be translocated to the host environment and directly interact with host proteins to exert virulence functions. Three host proteins were identified as putative targets of VdCUE through Y2H analysis, suggesting that VdCUE may regulate virulence by interacting with them.

Among these host targets, one is peptidyl-prolyl cis-trans isomerase (PPIase), and Cyclophilins (CYPs) are classified as members of the PPIase family. The *A. thaliana* genome encodes 31 putative CYPs (AtCYPs), which exhibit diverse biological functions including the responses to abiotic and biotic stresses [35]. In particular, *AtCYP19* and *AtCYP57* have been shown to play a vital role in plant immunity activation. Knockout of each led to increased susceptibility to *P. syringae* infection, and overexpression of *AtCYP19* led to increased reactive oxygen species (ROS) accumulation while overexpression of *AtCYP57* led to more callose deposition [36]. AtCyp18-3 and AtCyp19-3 could bind to p33, the viral replication protein of Tomato bushy stunt virus (TBSV), and inhibit virus replication [37]. In *Gossypium hirsutum*, *GhCYP3* contributes to the resistance against *V. dahliae* by suppressing the activity of GhPUB17, a negative regulator of cotton immunity [38].

Another host target is a 40S ribosomal protein. As core components of the ribosome, ribosomal proteins are primarily involved in protein biosynthesis, including mRNA and tRNA recognition, ribosomal subunit assembly, and maintenance of ribosomal stability. In addition, numerous studies have shown that certain ribosomal proteins contribute to plant disease resistance. As an anti-virus strategy, ribosomal protein RPL10A is phosphorylated by NIK1 kinase, enters the nucleus, and interacts with the putative transcription factor LIMYB, leading to the downregulation of global translation in host cells [39]. Potato 40S ribosomal protein S5 (StPRS5) has been shown to play a positive role in resistance to *Phytophthora infestans*, the causal agent of potato late blight [40]. *StPRS5* is transcriptionally induced by *P. infestans* infection, and overexpression of *StPRS5* increases reactive oxygen species (ROS) accumulation and disease resistance, making it a target of pathogen effectors to facilitate their virulence functions [40]. Similarly, cotton ribosomal protein S6 (GhRPS6) serves as a resistance factor against *V. dahliae*, functioning mainly in regulating the levels of SA, JA, and ROS [41]. Another cotton SA-responsive ribosomal protein, L18 (GaRPL18), also plays a positive role in resistance to *V. dahliae* [42]. In summary, ribosomal proteins play conserved roles in resistance against phytopathogens including viruses, oomycetes, and fungi.

The third host target of VdCUE is a tRNA-specific 2-thiouridylase. Thiouridylase is mainly responsible for tRNA thiolation, which is conserved in nearly all species, to allow translation efficiency and accuracy [43]. Cytosolic tRNA thiouridylase complex is essential for thiolation on tRNAs and maintaining genome integrity in *Schizosaccharomyces pombe* and *Caenorhabditis elegans* [44]. In plants, *Arabidopsis* cytosolic thiouridylase CTU2 is required not only for tRNA thiolation, but also for normal root development, and its deletion leads to a reduction in lateral root formation [45]. Thus, this target may also play a role in root development. As *V. dahliae* is a soil-borne pathogen that infects hosts from the roots, the interaction between VdCUE and this target (tRNA-specific 2-thiouridylase) may regulate root development to facilitate *V. dahliae* infection. Moreover, studies in recent years have revealed that tRNA thiolation enables efficient translation of *Arabidopsis* NPR1, a master regulator in the SA signaling pathway, thus regulating plant immunity [46].

Considering the importance of host target family members in disease resistance, VdCUE may regulate the virulence of *V. dahliae* by interacting with these host targets and modulating their biological functions. The CUE domain is known to exert a ubiquitin-binding function and is involved in ubiquitin chain formation during ERAD, which is essential for the degradation of a subset of substrates [47]. Based on this conserved function of the CUE domain, we hypothesize that the CUE domain in VdCUE may mediate the degradation of its disease-resistant host targets, which could in turn facilitate *V. dahliae* infection. This hypothesis is supported by precedents from other phytopathogens. For example, *Fusarium graminearum* orphan protein Osp24 targets host SNF1-related kinase TaSnRK1α and accelerates its degradation by promoting its interaction with the ubiquitin–26S proteasome system [48]. The secreted effector XopK from *Xanthomonas oryzae* ubiquitinates rice somatic embryogenic receptor kinase 2 (OsSERK2) and promotes its degradation via the 26S proteasome, thereby inhibiting pathogen-associated molecular pattern (PAMP)-triggered immunity [49]. However, whether VdCUE harbors ubiquitin-binding activity and modulates target degradation or biological functions through ubiquitination remains to be further investigated.

## Figures and Tables

**Table 1:** Screening of interacting proteins of VdCUE from *V. dahliae* XJ592 in cotton.

Bait Protein	Target Protein Gene ID	Functional Annotation
VdCUE	LOC107959305	tRNA-specific 2-thiouridylase
	LOC107953988	Peptidyl-prolyl cis-trans isomerase
	LOC107962302	40S ribosomal protein

## Data Availability

The data presented in this study are available within the article and the Supplementary Materials.

## Supplementary Materials

The following supporting information can be downloaded at https://www.mdpi.com/article/10.3390/jof11090660/s1, Figure S1: VdCUE positively regulates virulence and enhances the defoliating ability of *V. dahliae* XJ592; Figure S2: Expression of *VdCUE* in *N. benthamiana* fails to trigger plant cell death and does not interfere with BAX-mediated cell death; Table S1: Primers used in this research; Table S2: Gene and protein sequences of VdCUE and its ortholog VDAG_09947 (strain VdLs.17, assembly ASM15067v2).
