# Supplementary material for: Secreted Protein VdCUE Modulates Virulence of Verticillium dahliae Without Interfering with BAX-Induced Cell Death
Source: J Fungi (Basel). 2025 Sep 8;11(9):660. doi: 10.3390/jof11090660 (PMC12470305; doi:10.3390/jof11090660)
Supplement: Supplementary file 1 [file jof-11-00660-s001.zip › jof-3816440-supplementary/jof-3816440 Supplementary files/jof-3816440 Supplementary Figure.pdf]

Supplementary Figure 1

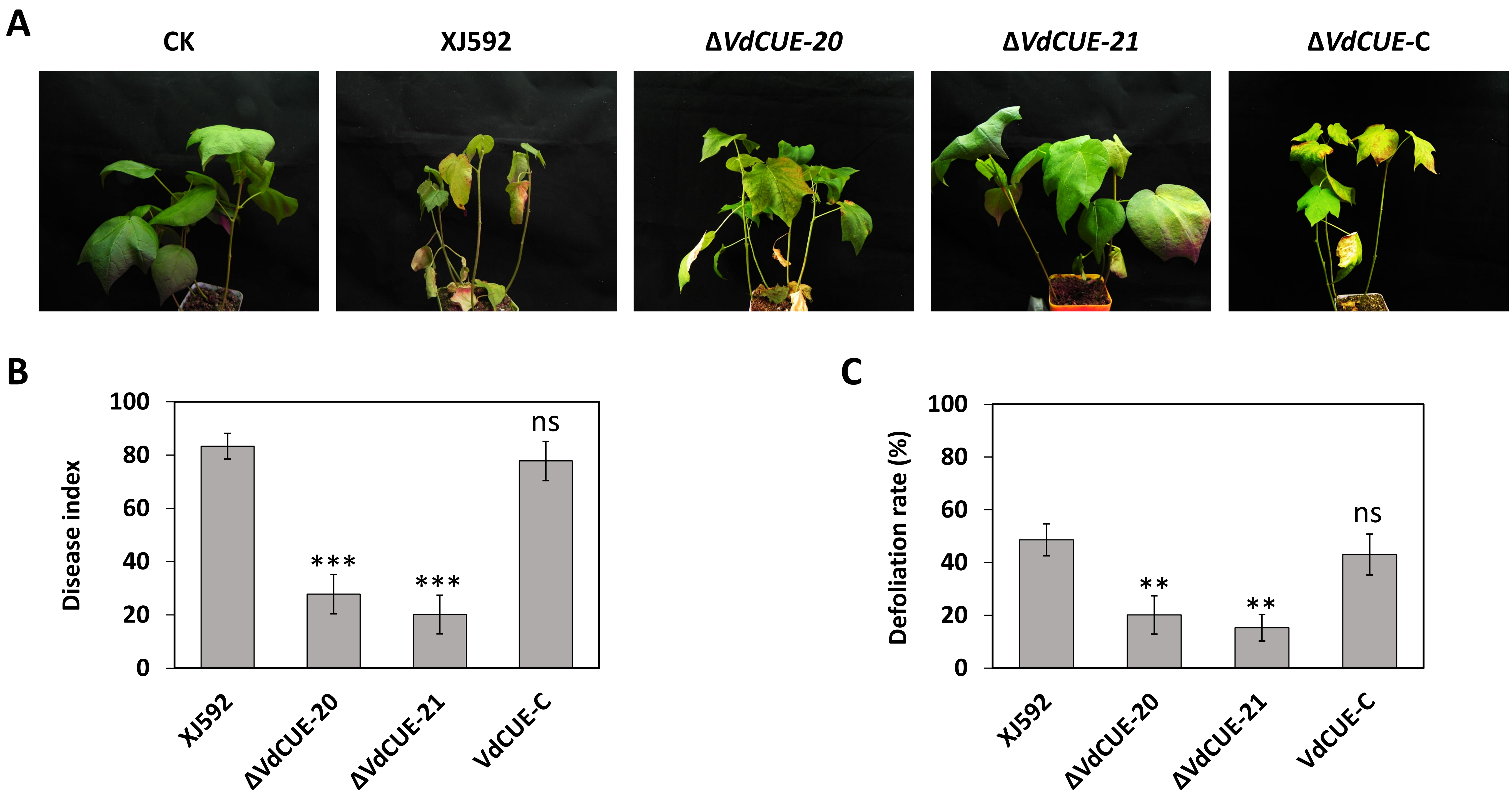

Figure S1. VdCUE positively regulates virulence and enhances the defoliating ability of *V. dahliae* XJ592.

Supplementary Figure 2

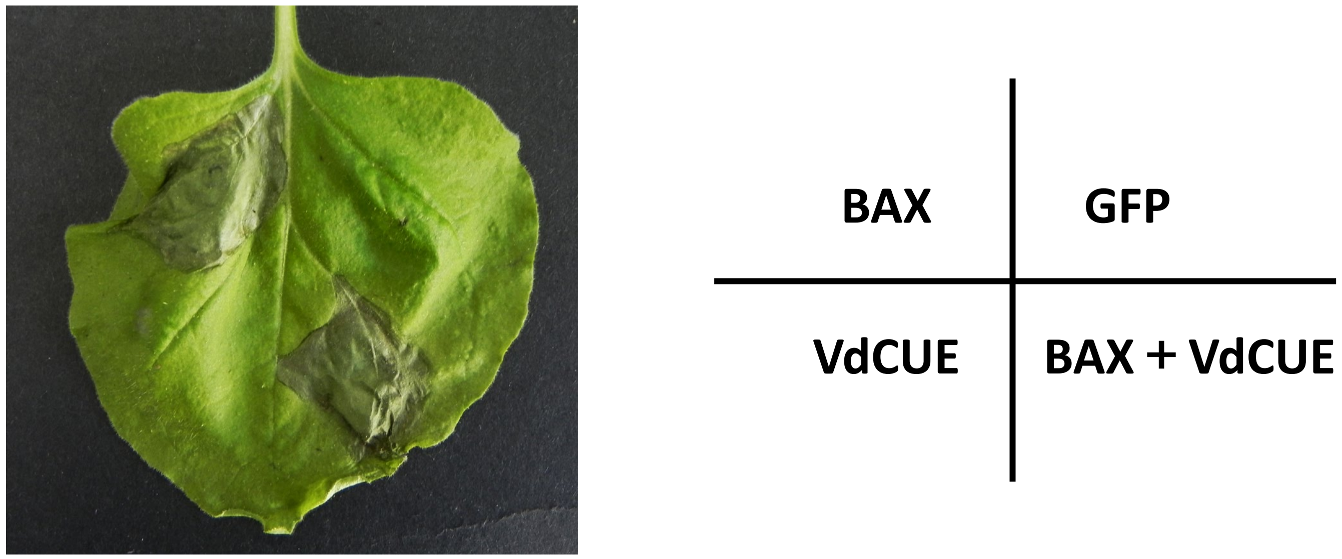

Figure S2. Expression of *VdCUE* in *N. benthamiana* fails to trigger plant cell death and does not interfere with BAX-mediated cell death.
