# Supplementary material for: Secreted Protein VdCUE Modulates Virulence of Verticillium dahliae Without Interfering with BAX-Induced Cell Death
Source: J Fungi (Basel). 2025 Sep 8;11(9):660. doi: 10.3390/jof11090660 (PMC12470305; doi:10.3390/jof11090660)
Supplement: Supplementary file 1 [file jof-11-00660-s001.zip › jof-3816440-supplementary/jof-3816440 Supplementary files/jof-3816440 Table S1 Primers used in this research.pdf]

Table S1. Primers used in this research.

Primer name	Sequence 5′-3′
pGKO-CUE-up-F	GAGCTCGCTGAGGGTTTAATTAAGTGTGCGGGAACGAGAATGTG
pGKO-CUE-up-R	ATGGGCCCGCTGAGGACTTAATTAAGAGGTGTGAGCGAAGTGAAGGA
pGKO-CUE-dn-F	ACTAGTGCTGAGGCATTAATTAACGCTCACAGTCGTCACAATCAG
pGKO-CUE-dn-R	AAGCTTGCTGAGGTCTTAATTAACGCCAACTTCCATCCGTTCATC
pBin-CUE-F	ACCCCCGGGGTCGACGGATCCATGGTTCATCGGTTACTCCGT
pBin-CUE-R	TCTAGTTCATCTAGAGGATCCACCAGGGAAGCCACCCCT
VdCUE-F	CGGCACCAACCCAGTCACAAAAC
VdCUE-R	CTCCTCCGAAAAGATGATGGAACCC
pSUL_tef_CUE_F1	AACCTCTAGAGGATCCGCCACCATGAACCCACCGCTCAGG
pSUL_tef_CUE_R	GCAGCTTCTGCGAATTCACCAGGGAAGCCACCCCT
HPT-3F	TCTCCTTGCATGCACCATTCCTTG
HPT-5R	AAATTTTGTGCTCACCGCCTGGAC
BK-VdCUE-F	ATGGCCATGGAGGCCGAATTCACCAGAAACACAGAAAGTACAGAAACTGG
BK-VdCUE-R	CCGCTGCAGGTCGACGGATCCACCAGGGAAGCCACCCCT
pSUC2-VdCUE-SP-F	CGGAATTTTAATTAAGAATTCATGAACCCACCGCTCAGG
pSUC2-VdCUE-SP-R	CACTATAGGGAGAACCTCGAGGGAATTGCCGTCTTCCGT
